# The Comparison of Advanced Electrospun Materials Based on Poly(-3-hydroxybutyrate) with Natural and Synthetic Additives

**DOI:** 10.3390/jfb13010023

**Published:** 2022-02-28

**Authors:** Polina Tyubaeva, Ivetta Varyan, Alexey Krivandin, Olga Shatalova, Svetlana Karpova, Anton Lobanov, Anatoly Olkhov, Anatoly Popov

**Affiliations:** 1 Academic Department of Innovational Materials and Technologies Chemistry, Plekhanov Russian University of Economics, 36 Stremyanny Per., 117997 Moscow, Russia; ivetta.varyan@yandex.ru (I.V.); avlobanov@mail.ru (A.L.); aolkhov72@yandex.ru (A.O.); popov.ana@rea.ru (A.P.); 2 Department of Biological and Chemical Physics of Polymers, Emanuel Institute of Biochemical Physics, Russian Academy of Sciences, 4 Kosygina Str., 119334 Moscow, Russia; a.krivandin@sky.chph.ras.ru (A.K.); shatalova@sky.chph.ras.ru (O.S.); karpova@sky.chph.ras.ru (S.K.)

**Keywords:** poly(3-hydroxybutyrate), porphyrin complex, hemin, tetraphenylporphyrin with iron, electrospun fibrous materials, molecular mobility, supramolecular structure, antibacterial effect

## Abstract

The comparison of the effect of porphyrins of natural and synthetic origin containing the same metal atom on the structure and properties of the semi-crystalline polymer matrix is of current concern. A large number of modifying additives and biodegradable polymers for biomedical purposes, composed of poly(-3-hydroxybutyrate)-porphyrin, are of particular interest because of the combination of their unique properties. The objective of this work are electrospun fibrous material based on poly(-3-hydroxybutyrate) (PHB), hemin (Hmi), and tetraphenylporphyrin with iron (Fe(TPP)Cl). The structure of these new materials was investigated by methods such as optical and scanning electron microscopy, X-ray diffraction analysis, Electron paramagnetic resonance method, and Differential scanning calorimetry. The properties of the electrospun materials were analyzed by mechanical and biological tests, and the wetting contact angle was measured. In this work, it was found that even small concentrations of porphyrin can increase the antimicrobial properties by 12 times, improve the physical and mechanical properties by at least 3.5 times, and vary hydrophobicity by at least 5%. At the same time, additives similar in the structure had an oppositely directed effect on the supramolecular structure, the composition of the crystalline, and the amorphous phases. The article considers assumptions about the nature of such differences due to the influence of Hmi and Fe(TPP)Cl) on the macromolecular and fibrous structure of PHB.

## 1. Introduction

One of the most effective ways to create binary compositions based on biocompatible polymers for biomedical purposes is electrospinning (ES) [1]. Electrospun materials have an extremely high specific surface area, which is a big advantage for biomedicine [2]. ES is a unique technique used for the effective introduction of various modifying additives of natural and synthetic origin [3]. The use of a large number of different additives makes it possible to solve many problems associated with obtaining materials with controlled properties and structure [4]. Of particular interest is the selection of such additives, which can significantly increase the characteristics of known biocompatible polymers, even at low concentrations.

Among different additives, close attention is paid to porphyrins, which a widely used in tumor and gene therapy [5,6,7], biomedicine [8,9], chemotherapy [10], and drug delivery [11]. Due to their chemical structure, porphyrins possess excellent chemical and thermal stabilities, photophysical and electrochemical performances, and biological compatibility [12]. Moreover, close attention should also be paid to the antimicrobial and antiviral activities of porphyrins [13]. In terms of the chemical origin and structural differences, two classes of porphyrins—natural and synthetic—can be distinguished [14].

Both of these classes are widely used in composition with biocompatible, biodegradable polymeric materials because of the ease of producing, simplicity, and lower investment costs compared to other nanoparticulate systems [15]. There is a wide variety of approaches to creating a porphyrin-polymer system: weak interactions, such as hydrophobic, electrostatic forces, coordination interaction, and hydrogen bonding [16].

Among a wide sample of polymer matrices, poly(3-hydroxybutyrate) (PHB) is of particular interest as a promising polymer for therapeutic applications. PHB is characterized by a high melting point, a high degree of crystallinity, and low permeability to oxygen, water, and carbon dioxide [17]. This bio-based polymer is biocompatible [18], obtained from renewable sources [19], and degrades in the biologically active environment [20].

There is a wide number of PHB-based composites biomaterials with poly(ethylene glycol) [21], polylactide [22], polycaprolactone [23], chitosan [24], elastomers [25], nanoparticles [26], carbon nanotubes [27], catalysts and enzymes [28], and bioactive molecules [29]. There are several works in which electrospun PHB-porphyrin composite materials were obtained: Polystyrene/Polyhydroxybutyrate/Graphene/Tetraphenylporphyrin [30], Polyhydroxybutyrate/Hemin [31], Polyhydroxybutyrate/Tetraphenylporphyrin with iron [32], and Polyhydroxybutyrate/5,10,15,20-*tetrakis*(4-hydroxy-phenyl)-21H,23H-porphine [33].

In a large number of works it was shown that PHB-based materials are immunologically inert, which allows using these materials as biocompatible for different biomedical applications [34,35,36,37]. Composites based on PHB and various additives did not cause any inflammatory reaction accompanied by leukocyte migration [38] and they had no hemolytic effect on the red cell suspension, so were they suitable for the blood-contacting applications [39].

The purpose of this work was to compare the effect of two porphyrins of natural and synthetic origin, containing the same metal atom in order to identify the possibilities of creating highly effective materials for biomedicine. As objects for polymer-porphyrin systems, the following hemin (Hmi) and tetraphenylporphyrin with iron (Fe(TPP)Cl) were selected.

Hmi is thermally stable [40], antimicrobial active against *Staphylococcus aureus* [41], biocompatible, and can be used for medical purposes [42,43]. Moreover, there are several successful works of creating electrospun materials with Hmi [44,45]. Materials containing Hmi can be successfully used for different biomedical applications [46], including the containers for drug delivery systems [47] because of their biocompatibility. The most important properties of hemin include the catalytic functions of heme and its oxidized form [48] and thermal stability [49]. Owing to the functionality of the porphyrin ring composed of a tetrapyrrole scaffold, Hmi can easily coordinate with many transition metal ions [48] and its redox properties could be easily controlled [50].

On the other hand, Fe(TPP)Cl is known for its catalytic effect [51,52]. This porphyrin complex is used in various fields of chemistry and biomedicine [53]. The magnetic properties of Fe(TPP)Cl are good enough for biomedical and therapeutic application [54]. It is antimicrobial active against Gram-positive Staphylococcus aureus and Gram-negative *Escherichia coli* [55]. Fe(TPP)Cl has low toxicity to eukaryotic cells [13]. Investigating the geometry of this complex allows us to significantly influence its electronic and electrical properties [51].

## 2. Materials and Methods

### 2.1. Materials

**Figure 1:**
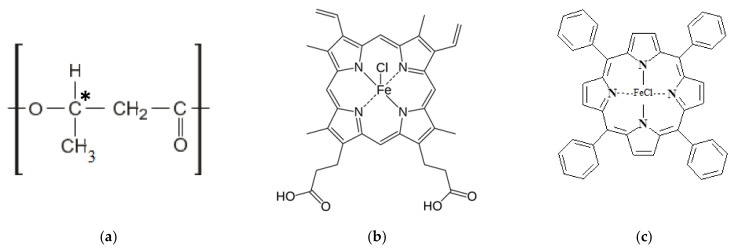
Structural formulas of PHB (**a**) [31], where * is the designation of the chiral carbon atom, Hmi (**b**) [31], Fe(TPP)Cl (**c**).

Poly(3-hydroxybutyrate) (PHB) 16F series (BIOMER, Frankfurt am Main, Germany) with molecular weight of 206 kDa, density of 1.248 g/cm^3^, and crystallinity of 59% was used (Figure 1a) as a polymeric matrix. Hemin (Hmi) isolated from bovine blood (Moscow, Russia) was used as a modifying additive of natural origin (Figure 1b) [56]. Tetraphenylporphyrin with iron (Fe(TPP)Cl) (Moscow, Russia) was used as a modifying additive of synthetic origin (Figure 1c) [56]. Both tetrapyrroles are coordination complexes of iron (oxidation state: III) [57].

### 2.2. Preparation of the Electrospun Materials

Polymer nanofibrous materials based on PHB-porphyrin composition were obtained by electrospinning method (ES) on a single-capillary laboratory unit EFV-1 (Moscow, Russia). A photo and schematic view of the laboratory unit are shown in Supplementary Materials Figure S6. The diameter of the capillary was 0.1 mm. The collector was stable, 300 × 300 mm. The consumption of the forming solution was 5–7 × 10^−5^ g/s.

The PHB forming solution was prepared by dissolving the well-dispersed powder in chloroform at 60 °C. The content of PHB was 7% wt. The electrical conductivity of the 7% PHB in chloroform was 8 μS/cm and the viscosity was 1.0 Pa s.

The PHB-Fe(TPP)Cl materials were obtained from the forming solution with a content of Fe(TPP)Cl—1, 3, and 5% wt. of the PHB. Fe(TPP)Cl was dissolved in chloroform in the PHB solution at 25 °C. The electrical conductivity of the PHB-Fe(TPP)Cl forming solution was 12–15 μS/cm and viscosity was 1.0–1.2 Pa s [32]. The voltage of the ES for the PHB-Fe(TPP)Cl solution was 17–19 kV, the distance between the electrodes was 200–210 mm, and the gas pressure on the solution was 5–10 kg(f)/cm^2^.

The PHB-Hmi materials were obtained by the method of double-solution electrospinning [58,59]. Hmi was dissolved in N,N-dimethylformamide at a temperature of 25 °C and homogenized with the PHB solution. The electrical conductivity of the forming solution was 10–14 μS/cm and the viscosity was 1.4–1.9 Pa s [31]. The voltage of the ES for the PHB-Hmi solution was 19–20 kV, the distance between the electrodes was 210–220 mm, and the gas pressure on the solution was 5–7 kg(f)/cm^2^.

### 2.3. Methods

#### 2.3.1. Microscopy

Primary data of morphology and topology of the fibrous materials with different content of Fe(TPP)Cl and Hmi was obtained by optical microscope Olympus BX43 (Tokyo, Japan) and Scanning electron microscopy (SEM) by Tescan VEGA3 microscope (Wurttemberg, Germany) in order to characterize the morphology. Optical microphotographs were obtained in the reflected light at a magnification of 200 times. The SEM microphotographs were obtained at an accelerating voltage of 20 kV at a magnification of 500 times. PHB-Fe(TPP)Cl and PHB-Hmi samples 10 × 10 mm were covered with a platinum layer for the SEM method.

#### 2.3.2. Morphology and Density Analysis

The structure of fibrous materials was evaluated by the counting method using the software Olympus Stream Basic (Tokyo, Japan). PHB-Fe(TPP)Cl and PHB-Hmi samples 100 × 100 mm were used for the counting method.

The average diameter of fibers was determined manually for each fiber on the z-stack at 10 different points of the sample excluding defective areas.

Average density characterizes the mass per unit volume of the material. The data were averaged over 10 samples. The density, *δ,* was defined as:

(1) $$\delta =\frac{m}{lxBxb},$$

where *m* is the mass; *l* is the length; *B* is the width; and *b* is the thickness.

Theoretical porosity is the percentage of the mass of the material and the fiber-free volume. The data were averaged over five samples.

#### 2.3.3. Differential Scanning Calorimetry

Thermal properties of the PHB-Fe(TPP)Cl and PHB-Hmi samples were studied using differential scanning calorimeter (DSC) by Netzsch 214 Polyma (Selb, Germany), in an air atmosphere, with a heating rate of 10 °K/min and with a cooling rate of 10 °K/min. The results of scanning in the air atmosphere corresponded to the values of scanning in the argon atmosphere due to the low oxidation of the samples. The samples weight was 6–7 mg. The samples were heated from 20 °C to 220 °C and then cooled to 20 °C twice. The average statistical error in measuring thermal effects was ±2.5%.

Enthalpy of melting, Δ*H*, was calculated by NETZSCH Proteus software according to the standard technique [60].

Crystallinity degree, *χ*, was defined from the melting peak as:

(2) $$\chi =\frac{\Delta H}{H_{PHB}}\times 100\%,$$

where Δ*H* is the melting enthalpy; *H_PHB_* is the melting enthalpy of the ideal crystal of the PHB, 146 J/g [61]; *C* is the content of the PHB in the composition.

#### 2.3.4. Electron Paramagnetic Resonance

The state of the amorphous phase of PHB in the polymer matrix was studied using electron paramagnetic resonance (EPR) by EPR-V automatic spectrometer (Moscow, Russia). The modulation amplitude was < 0.5 G. The spin probe was 2,2,6,6-tetramethylpiperidine-1-oxyl (TEMPO). TEMPO was introduced into the samples from the gas phase at 50 °C.

Radical concentration in the polymer was determined by the Bruker WinEPR software (the reference was CCl4 with the radical concentration not exceeding 10^–3^ mol/L). The average statistical error in measuring thermal effects was ±5%.

The experimental spectra of the spin probe in the region of slow motions (τ > 10^–10^ s) were analyzed within the model of isotropic Brownian rotation using the program described in [62]. Probe rotation correlation time, τ, in the region of fast rotations (5 × 10^–11^ < τ < 10^–9^ s) was found based on the ESR spectra from the formula [63]:

(3) $$\tau =\Delta H+\times (\sqrt{\frac{I_{+}}{I_{-}}}\;–1)\times 6.65\times 10{{–}^{10}}^{,}$$

where Δ*H_+_* is the width of the spectrum component located in a weak field and $\frac{I_{+}}{I_{-}}$ is the ratio of the component intensities in the weak and strong fields.

#### 2.3.5. X-ray Diffraction Analysis

The state of the crystalline phase of PHB in the polymer matrix was studied by X-ray diffraction analysis. The intensity of wide- and small-angle X-ray scattering was measured in transmission geometry on a diffractometer with optical focusing of the X-ray beam and a linear coordinate (position-sensitive) detector [64,65] (X-ray tube with a copper anode, Ni filter) and was corrected for background scattering. The intensity of wide-angle X-ray scattering was also measured in Bragg–Brentano reflection geometry on an HZG4 diffractometer (Freiberger Präzisionsmechanik, Germany) with a diffracted beam graphite monochromator (CuKα radiation).

Degree of crystallinity of PHB, *ξ*, was calculated with the diffraction patterns obtained in transmission geometry, as [66]:

*ξ* = (*I_exp_* − *I_am_*)/(*I_exp_* − *I_b_*) ×100% (4)

where *I_exp_* is the integral experimental intensity of the diffractogram of the sample; *I_am_* is the integral intensity of the hypothetical diffractogram of the amorphous phase passing through the points of minima between the diffraction maxima, *I_b_* is the integral intensity of a baseline underlying experimental diffractogram.

Average sizes of PHB crystallites were calculated from diffractograms obtained with the Bragg-Brentano method using the Selyakov-Scherrer formula as:

*L_hkl_* = *λ*/(*β_hkl_* ∙ cos *θ_hkl_*), (5)

where *L_hkl_* is the average size of the crystallites calculated with the diffraction line (hkl), λ is the wavelength of X-ray radiation, *β_hkl_* and *θ_hkl_* are the integral width (in radians on the 2θ scale) corrected for instrumental broadening and half of the scattering angle for the diffraction line (hkl), respectively.

#### 2.3.6. Mechanical Analysis

Mechanical properties were examined by a tensile compression testing machine Devotrans DVT GP UG 5 (Istambul, Turkey). The stretching speed was 25 mm/min. The preload pressure was absent. PHB-Fe(TPP)Cl and PHB-Hmi samples size were 10 × 40 mm. The data were averaged over five samples.

Tensile strength was registered automatically by Devotrans software. The average statistical error in measuring thermal effects was ±0.02 MPa.

Elongation at break, *ε*, was calculated as:

(6) $$\varepsilon =\frac{\Delta l}{l_{0}}\times 100\%,$$

where Δ*l* is the difference between the final and initial length of the sample; *l_0_* is the initial length of the sample. The average statistical error in measuring thermal effects was ±0.2%.

#### 2.3.7. Wetting Contact Angle Measurement

The wettability and degree of hydrophilicity of the surface of the PHB-Fe(TPP)Cl and PHB-Hmi samples were evaluated by measuring the contact angle of wetting formed between a drop of water and the surface of the sample. Water drops (2 µL) were applied to three different areas of the film surface by an automatic dispenser.

The marginal wetting angle of the surface of the samples was measured using an optical microscope M9 No. 63649, lens FMA050 (Moscow, Russia). Image processing was done using Altami studio 3.4 software. The result is the average of three measurements from different parts of the sample. The relative measurement error was ±0.5%

#### 2.3.8. Biological Analysis

The antimicrobial activity of PHB-Fe(TPP)Cl and PHB-Hmi samples was studied by biomedical tests on cellular material. *Staphylococcu. aureus p 209, Salmonella. typhimurium,* and *Escherichia coli 1257* were used as test cultures. Samples of initial PHB were served as a control. Cultures of test microorganisms were transplanted onto meat-peptone agar and incubated for 24 h at 37 °C. Then, a suspension of each microorganism was prepared in saline solution and the concentration of microbial cells was determined according to the turbidity standard of 104 mk/mL. PHB-Fe(TPP)Cl and PHB-Hmi samples size were 20 × 20 mm. Samples were placed in sterile Petri dishes, to which 1 mL of a test culture suspension was added and kept at room temperature for 30 min. After that, 10 mL of sterile saline solution was poured into the cup and kept for 15 min to elute the test culture from the samples of the test material. After the exposure, the suspension from the cups in the amount of 100 mL was sown on the surface of meat-peptone agar, previously poured into Petri dishes. The crops were incubated for 48 h at 37 °C. In parallel, the test culture suspensions used in the experiment were seeded to control the concentration of viable microorganisms. Then, the colonies of viable microorganisms grown on the surface of the agar were counted.

## 3. Results and Discussion

Poly(-3-hydroxybutyrate) has many advantages, which are enhanced by the development of the electrospinning method: the material degrades rapidly in the soil, remaining stable in the air [67]; the fibrous structure compensates for the fragility of the semicrystalline polymer [68]; it is possible to introduce modifying additives evenly into the fiber’s structure [31]. Therefore, three levels of structural organization for electrospun materials could be distinguished: macroscopic (whole system), mesoscopic (fiber contact area), and microscopic (structure of the fiber) scale [69].

### 3.1. Electrospun Material Structure

To describe the morphology and mutual orientation of fibrous materials, it is convenient to use parameters that reliably characterize it: density; average diameter of the fibers; and porosity [31]. The formation of a unique highly developed structure of electrospun materials is influenced by a complex of parameters and depends on the type of polymer solution, processing parameters, and environmental conditions [70]. The introduction of additives into the polymer solution made it possible to significantly affect electrical conductivity, viscosity (which also affected the voltage), flow rate, Taylor’s cone shape, and the evaporation rate of the solvent. All these aspects had a great impact on the appearance of the produced fibers: color, surface character, morphology, the presence of inclusions, and defects.

#### 3.1.1. Optical Microscopy

**Figure 2:**
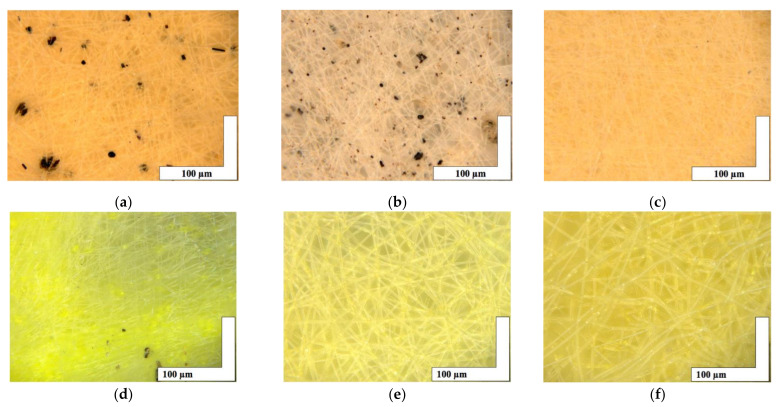
The microphotographs of PHB with different content of Hmi [31]: 1% wt. (**a**), 3% wt. (**b**) and 5% wt. (**c**) and Fe(TPP)Cl: 1% wt. (**d**), 3% wt. (**e**) and 5% wt. (**f**).

The microphotographs of the material based on the PHB with a different content of hemin and tetraphenylporphyrin with iron are shown in Figure 2.

The introduction of small concentrations (1, 3, and 5% wt. of PHB) of porphyrins of natural and synthetic origin containing a trivalent iron atom had a significant effect on the formation of the fibrous layer. Characteristics of the fibrous layer are presented in Table 1. It is important to note the formation of black inclusions 4–32 µm for 1% wt. of Hmi, 0.7–17 µm for 3% wt. of Hmi and 1% wt. of Fe(TPP)Cl. In the case of 5% wt. of Hmi (Figure 2c), inclusions are practically absent, their size was 1–4 µm. And in the case of 3 and 5% wt. of Fe(TPP)Cl (Figure 2e,f) inclusions are completely absent.

Table 1 shows that the presence of additives leads to a decrease in the density of the material: porosity increases markedly by 9–15% depending on the additive, and the density decreases by an average of 30–45% depending on the additive. At the same time, the trend of density changes coincides for both PHB-Hmi and PHB-Fe(TPP)Cl, but the trend of the change in the average size of the fibers differs significantly. The addition of Hmi leads to a decrease in the average diameter of the fibers by 42–50%. The addition of 1% wt. of Fe(TPP)Cl leads to a decrease in the average diameter of the fibers by 40% and the addition of 3 and 5% wt. of Fe(TPP)Cl leads to a small increase in the average diameter of the fibers by 1–2%, which is an extremely small impact.

#### 3.1.2. Scanning Electron Microscopy

**Figure 3:**
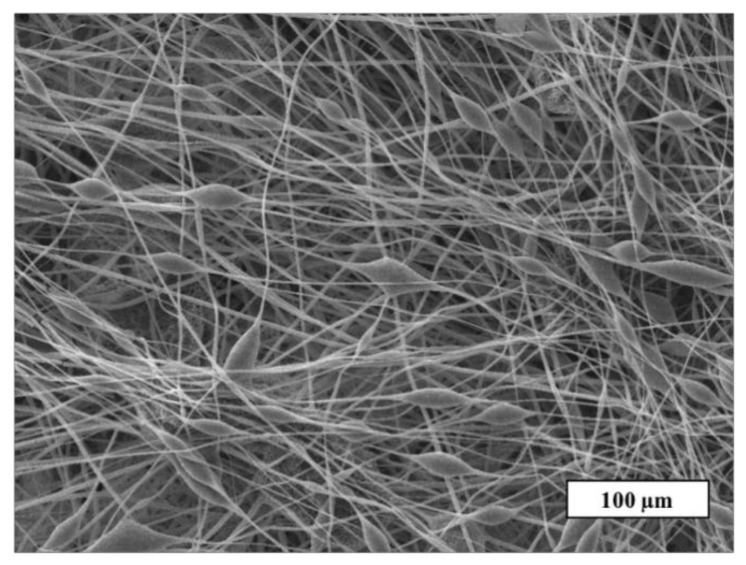
The SEM microphotograph of PHB electrospun material.

A detailed study of the fiber’s surface was carried out by the SEM method. The SEM microphotograph of the material based on the initial PHB is shown in Figure 3.

Figure 3 shows that the initial PHB electrospun material is characterized by a large number of defects: thickenings, fiber irregularities, and gluings. There are areas where the fibers are unevenly distributed or glued together. The size of the pear-shaped thickenings is 14–25 µm in diameter and their length is 20–70 µm on average. Such defects are mainly due to the insufficient balance of viscosity and electrical conductivity of the polymer forming solution [32]. Imbalanced polymer solution unevenly passes through the capillary, forming local thickenings on the surface of the fibers. Such fibers do not have time to fully cure at the stage of movement from the capillary to the collector, as a result of which glues and individual thickenings are formed. It is important to note that such areas can negatively affect the mechanical and diffusion properties. Moreover, such areas make the properties of the whole material inhomogeneous along the surface of the web, preventing reliable prediction of operational characteristics. Based on this, the effect of the porphyrins considered on the morphology of PHB electrospun materials can be assessed as positive.

**Figure 4:**
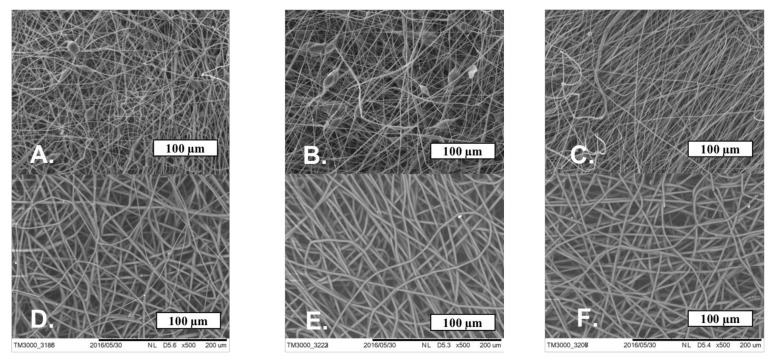
The microphotographs of PHB with a different content of Hmi: 1% wt. (**A**), 3% wt. (**B**) and 5% wt. (**C**) and Fe(TPP)Cl: 1% wt. (**D**), 3% wt. (**E**) and 5% wt. (**F**).

The SEM microphotographs of the material based on the PHB with different content of hemin and tetraphenylporphyrin with iron are shown in Figure 4.

Figure 4 shows that Hmi and Fe(TPP)Cl contribute to the formation of smooth uniform fibers, reducing the number of defects. With the introduction of 1 and 3% wt. of Hmi, the number of defects and glues is reduced by 50–60%, and the introduction of 5% wt. of Hmi allowed to obtain completely faultless fibers. The thickness and tortuosity of the fibers decreased with an increase in the concentration of Hmi, which has a positive effect on the uniformity of operational properties. Fe(TPP)Cl in all concentrations had a strong effect on the fibrous structure, making the fibers uniform and smooth without snagging and thickening. A total of 1% wt. of Fe(TPP)Cl allowed obtaining thinner fibers than 3 and 5% wt. of Fe(TPP)Cl, which differed little from each other. However, those fibers (Figure 4D) were characterized by a large thickness difference with local thickness distinctness of 70–75%.

### 3.2. Supramolecular Structure

Semi-crystalline polymers have a metastable structure, where various nanophases can be crystalline, liquid, glass, or mesophase. This multi-level structure is installed during material processing [71]. In biomedical applications, the supramolecular structure plays a significant role in the degradation, stability, and properties of the final product [70]. The predicted control of different parameters of the supramolecular structure is very important for constructing biomedical material with regulated properties.

The supramolecular structure of PHB is well known. PHB crystallizes into α-form crystal modification from the melt, which has an orthorhombic unit cell with *a* = 5.76 A°, *b* = 13.20 A°, *c* (fiber axis) = 5.96 Å [72]. Crystallites of PHB tend to be laid in lamellae [73] and PHB spherolites are possible in case of sufficient time and optimal conditions for repeated cold crystallization [74]. The ES process promotes rapid curing of the dissolved polymer. The polymeric fibers are fixed in the material, having a predominant orientation if an optimal balance of electrical conductivity, viscosity, and molding conditions is found for the polymer [75].

#### 3.2.1. X-ray Diffraction Analysis

**Figure 5:**
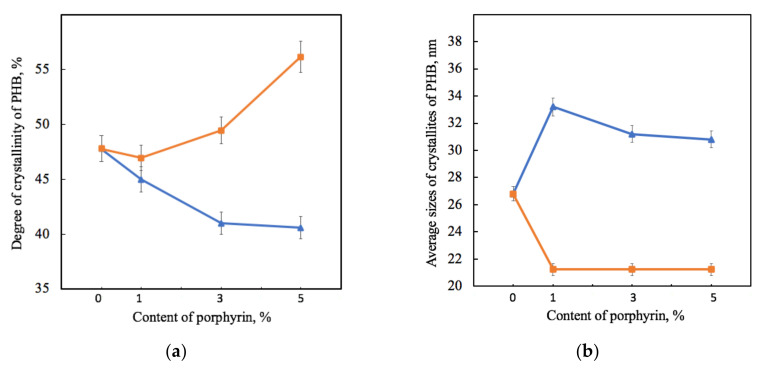
Dependence of the degree of crystallinity (**a**) and average sizes of PHB crystallites *L_020_* (**b**) on the amount of porphyrin according to X-ray diffraction analysis for PHB-Hmi (blue) and PHB-Fe(TPP)Cl (orange).

By X-ray diffraction analysis, it was found that the introduction of additives did not affect the parameters of the orthorhombic crystal lattice of PHB (*a* = 0.576 nm, *b* = 1.320 nm, *c* = 0.596 nm, space group symmetry of P2_1_2_1_2_1_). The values of the long period were close to each other, which were between 5.2 and 5.4 nm for samples containing Hmi and between 5.7 and 5.9 nm for samples containing Fe(TPP)Cl. As a result of the analysis of X-ray diffractograms, the values characterizing the crystalline phase of PHB were obtained (Figure 5). X-ray diffractograms are shown in Supplementary Materials Figures S2 and S3 for the PHB-Hmi system and X-ray diffractograms for the PHB-Fe(TPP)Cl system were discussed in previous work [32].

Figure 5 shows that the effect of Hmi and Fe(TPP)Cl on PHB was significantly different. The gradual decrease in the proportion of crystallites of PHB-Hmi samples was between 6 and 17%, and the gradual decrease in the proportion of crystallites of PHB-Fe(TPP)Cl samples was between 3 and 17%. Addition of 1% wt. of Fe(TPP)Cl has a low effect on the degree of crystallinity of PHB. The introduction of Hmi contributes to a slight increase in the size of PHB crystallites by 14–20%, while the introduction of Fe(TPP)Cl leads to a decrease in their size by 40% (Figure 5b). It should be mentioned that the longitudinal size of PHB crystallites L020 in PHB-Fe(TPP)Cl did not change with different content of additive (Figure 5b). However, at the same time the transverse size of the PHB crystallites L002 in PHB-Fe(TPP)Cl increased from 9.8 (0 and 1% wt.) up to 12.7 nm (3% wt.) and 12.5 nm (5% wt.).

#### 3.2.2. Electron Paramagnetic Resonance Analysis

The EPR method was used for characterizing the amorphous phase. EPR spectra of the spin probe TEMPO in structure of samples PHB-Hmi and PHB-Fe(TPP)Cl are shown in Supplementary Materials Figure S4.

**Figure 6:**
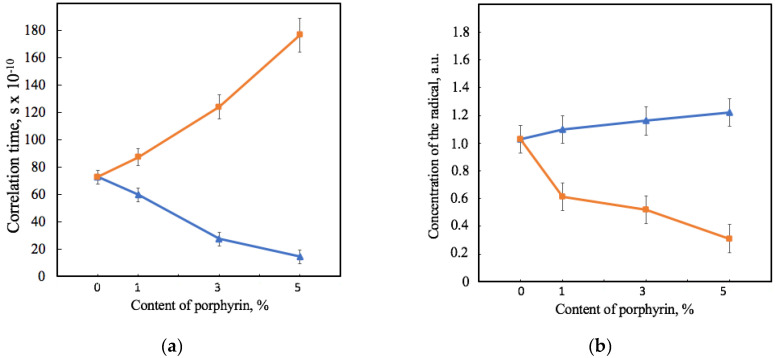
Dependence of the correlation time of the spin robe TEMPO in the structure of the samples (**a**) and the concentration of the spin probe in relation to the mass of the material’s sample (**b**) on the amount of porphyrin according to EPR analysis for PHB-Hmi (blue) and PHB0-Fe(TPP)Cl (orange).

Figure 6 shows that the effect of Hmi and Fe(TPP)Cl on PHB’s amorphous region was consistent with the effects shown previously by the X-ray diffraction method. The correlation time of the probe in PHB-Hmi samples decreases. TEMPO mobility becomes less by 18–80%. At the same time in the same conditions the correlation time of the probe in PHB-Fe(TPP)Cl samples increases by 19–140% (Figure 6a). These results are related to the concentration of the radical entering the samples of the material (Figure 6b). Fe(TPP)Cl most likely occupies space in the amorphous phase and prevents the penetration of the radical into the material, reducing the concentration of the radical by 40–70%. At the same time, Hmi does not prevent the penetration of TEMPO into the amorphous region. The more of the radical enters, the less mobility it has in the PHB-Hmi composition. The main reason for such an effect is the localization of Hmi in the amorphous phase, as there are no obstacles filling it with a radical.

#### 3.2.3. Differential Scanning Calorimetry Analysis

The structure of many semi-crystalline polymers, including PHB, cannot simply be described by a conventional two-phase model consisting of crystalline and amorphous phases [76]. Decoupling between the crystalline and amorphous phases is generally incomplete due to the length of the polymer molecules, which far exceeds the dimensions, at least in one direction, of the crystalline phase, and due to possible geometric limitations [77]. The intermediate phase is non-crystalline and includes amorphous sections of macromolecules, whose mobility is hindered by near-crystalline structures [77].

The DSC method is an effective instrument for studying whole crystalline structure including even near-crystalline structures, which melt at temperatures, close to the melting temperature of PHB crystallites. DSC thermograms of PHB-Hmi and PHB-Fe(TPP)Cl are shown in Supplementary Materials Figure S1.

**Figure 7:**
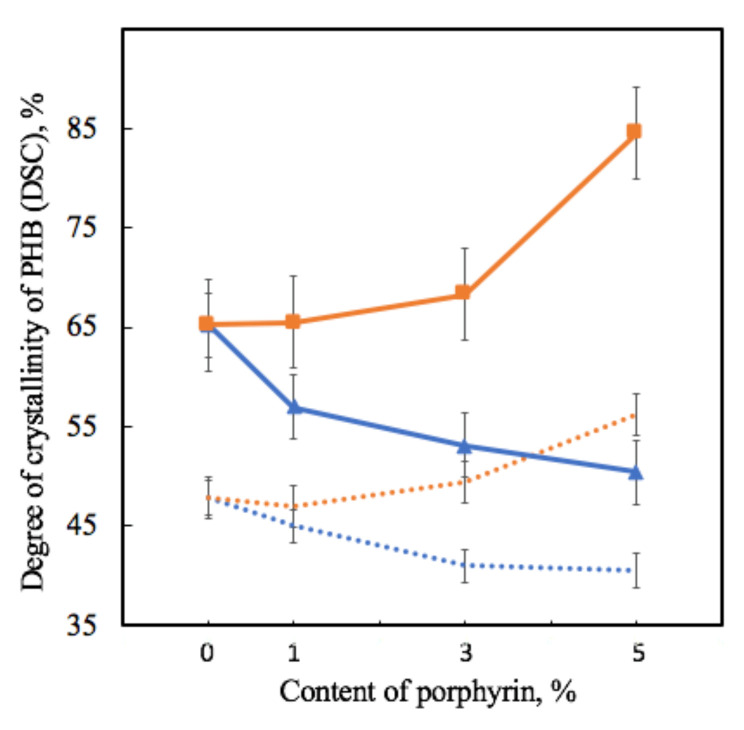
Dependence of the degree of crystallinity of samples on the amount of porphyrin according to DSC analysis for PHB-Hmi (blue line) and PHB-Fe(TPP)Cl (orange line) and X-ray diffraction analysis for PHB-Hmi (blue dots) and PHB-Fe(TPP)Cl (orange dots).

Table 2 shows that the melting temperature changes very slightly, with 3–6 °C, which is consistent with the assumption that PHB crystallites do not change much in size, since it significantly depends on this parameter. Of great interest are the values of crystallinity, with trends fully consistent with the results of the X-ray diffraction method (Figure 7).

As the degree of crystallinity in DSC is understood as the total fraction of the crystalline phase in a semi-crystalline polymer, which includes both well-crystallized crystallites and uncrystallized, defective and paracrystalline formations, it is seen that crystallinity in DSC decreases by 13–25% for PHB-Hmi and increases by 2–28% for PHB-Fe(TPP)Cl at the first heating.

### 3.3. Properties of Electrospun Materials

#### 3.3.1. Mechanical Analysis

The physical and mechanical properties of composite materials are an important class of operational properties, but they are also to a large extent an indicator of the state of the polymer-additive molecular system. The results of the mechanical tests are shown in Table 3. Stress-strain curves are shown in Supplementary Materials Figure S5.

Table 3 shows that high results in improving physical and mechanical properties were provided by 5% wt. of Hmi and 1% wt. of Fe(TPP)Cl. All other combinations of additives led to a reduction of the mechanical parameters of the material.

Mechanical properties are complex characteristics that depend on all levels of organization of nonwoven fibrous material. The contribution is made by defects of the fibrous layer, fiber bondings, and features of the supramolecular structure. There are two components that cause the growth of physical and mechanical characteristics. Firstly is the contribution of porphyrin with an atom of the metal to the formation of well-cured fibers without defects. These fibers form the layer with a higher possibility of withstanding loads due to the mobility of fibers in the whole system. Secondly, the addition of the porphyrin complexes affect the crystallization process, which can lead to a greater flexibility of the amorphous phase in the fiber and is capable of compensating for the high fragility of the initial PHB.

#### 3.3.2. Wetting Contact Angle Analysis

**Figure 8:**
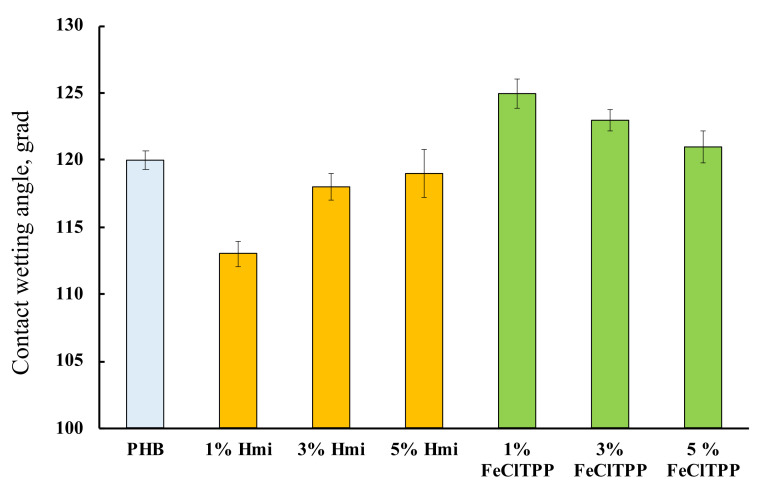
Contact wetting angles of the fibrous materials with a different amount of additives.

Wetting contact angles of the fibrous materials were determined to evaluate the hydrophobicity of the surface area. The results are shown in Figure 8.

PHB is hydrophobic [78]. This property persists after the ES process. The introduction of porphyrin complexes allowed to influence this property of the material to some extent. Interestingly, in both cases, the greatest effect was found for 1% of the additive, regardless of its nature.

#### 3.3.3. Biological Analysis

Biological tests made it possible to test the effectiveness of Hmi and Fe(TPP)Cl against Gram-positive and Gram-negative cultures. The results of the biological analysis tests are shown in Table 4.

The antibacterial properties of the Hmi and Fe(TPP)Cl are similarly high. In the PHB-Hmi and PHB-Fe(TPP)Cl, porphyrins are primarily associated with their effect on the cell walls of microorganisms by changing the charge of the bacterial cell. As a consequence, porphyrin molecules can suppress the function of adhesion and colonization of pathogens. Apparently, metal complexes are capable of disrupting the ionic balance of a living cell. In particular, this effect is enhanced in nanoscale fibrous materials. In addition, complexes containing metals of variable valences, such as iron, stimulate the formation of reactive oxygen species in aqueous media, which in turn also negatively affects the viability of pathogenic microorganisms. In general, the data obtained suggest that in the case of creating an antimicrobial material for biomedical purposes, the inclusion of Hmi or Fe(TPP)Cl in the composition positively affects the ability of the material to suppress the viability of bacteria and fungi.

## 4. Discussion

At the stage of the ES process, the introduction of both modifying additive at low concentrations (1, 3, and 5% wt.) can have a significant effect on the formation of the material’s structure. The addition of Hmi to the forming solution of PHB increases viscosity by 40–90% and electrical conductivity by 25–75%. These parameters let the jet of forming solution move fast enough to form regular uniform fibers during the ES process. As a result, the optimum of balance viscosity-electrical conductivity is obtained at a concentration of 5% wt. of Hmi (Figure 4C). The addition of Fe(TPP)Cl to the forming solution of PHB increases viscosity by 20% and electrical conductivity by 50–87%. As a result, the optimum of balance viscosity-electrical conductivity is obtained at a concentration of 1, 3, and 5% wt. of Fe(TPP)Cl (Figure 4D–F). Both additives provide such an effect, mostly because of the atom of the metal in its structure (Figure 1b,c), and serves as a good current conductor to ensure higher efficiency of the ES process.

Hmi and Fe(TPP)Cl contribute to the reduction of the density of the fibrous layer by 34–44% and by 30–47%, respectively, increasing the porosity no less than by 9%, which is a great advantage for producing material with a higher developed surface area. The change in average diameters occurs differently, but the main aspect is reducing the number of caverns, pear-shaped defects, smudges, and other negative consequences of insufficient forming properties of the forming solution.

Of interest are the black inclusions found on the surface of the 1 and 3% wt. of Hmi on the surface PHB-Hmi and 1% wt. of Fe(TPP)Cl on the surface PHB-Fe(TPP)Cl. In previous work [31] EDX elemental analysis showed that these inclusions were particles of Hemin. A lower concentration of Hmi and Fe(TPP)Cl leads to the formation of agglomerates during the curing of the solution. They can diffuse onto the surface of the fibers with a high probability, due to their small size, sticking together into larger formations. With an increase in their concentration, agglomerates of particles remain in the structure of the polymer fiber, discovered using X-ray diffraction, which showed the presence of a large-crystalline phase of hemin (crystallite sizes more than 50–100 nm) in 5% wt. of Hmi.

These changes are closely related to the supramolecular structure of PHB [79]. PHB preserves the order of the crystal lattice. At the same time, we see noticeable changes in the structure of the crystalline phase.

PHB macromolecules could be considered as the alternation of sections of the crystalline and amorphous phases that affect a number of physical and mechanical properties of PHB fibers [80]. The addition of the additives leads to an introduction of the crystallization centers into the polymer system. These centers allow macromolecules of PHB to take an advantageous position, which lets the crystalline phase form better organized structures. For both additives, the realization of this effect is observed. In the case of Hmi, the crystallites have a larger size with a smaller number of them (Figure 5). In the case of Fe(TPP)Cl, the crystallites have a larger number and a smaller size (Figure 5). This may indicate the different nature of the interaction of porphyrin particles with each other during the formation of a PHB fiber from a solution.

A significant increase in the degree of crystallinity of PHB (detected by X-ray method) in the system PHB-Fe(TPP)Cl was accompanied by a significant increase in the number of irregular crystal formations that contributed to the DSC signal. It must be the structures that most likely affect the decrease in physical and mechanical properties of the material. Such a large proportion of the crystalline phase leads to embrittlement of the fibers, despite the fact that they have fewer defects compared to the initial ones and a more refined morphology.

These assumptions are in good agreement with the EPR results. An increase in the number of crystallites leads to a decrease in the proportion of a loose and mobile amorphous phase, into which a radical can enter and rotate. In the case of Fe(TPP)Cl, the diffusion of the radical into the amorphous phase was hindered, most likely by particles of tetrapyrrole rings, which could most likely occupy the free volume of the amorphous phase (Figure 6). At the same time, Hmi contributes to the free rotation of the radical and its greater concentration in the fiber structure (Figure 6). This effect increases with the increase of the Hmi concentration. Considering the detected Hmi crystal formations, they are most likely localized at the boundary of the crystalline phase of the PHB or in the zones of rigid amorphous areas.

Hmi and Fe(TPP)Cl are thermally stable, so they have no contribution to the melting behavior of PHB, except for their important role in the formation of the supramolecular structure. The first heating shows the state of the PHB structure as a consequence of the ES process, while the second heating shows the initial polymer structure. This explains the decrease in temperature and enthalpy of melting by 3–5% on average for both. Each subsequent heating leads to a decrease in the fraction of regular crystallites that have managed to crystallize well even during fast cooling (10 °K/min during 20 min), converting poorly crystallized areas into a low-molecular fraction. Changes in the supramolecular structure and in the morphology and surface of fibers significantly affect the properties of materials.

The wetting angle changes slightly with the introduction of additives, however, hydrophobic PHB, most likely due to a change in the state of the surface, is slightly hydrophilized by 3–5% with the introduction of Hmi. The effect of Hmi should be due to the polar groups –COOH (Figure 1b) located in the structure of the tetrapyrrole ring. This can be a notable advantage for planning certain types of biomedical materials. At the same time, Fe(TPP)Cl slightly decreases the contact wetting angle by 2–4%, which leads to an increase in the hydrophobicity of the material. The absence of polar groups and the localization of Fe(TPP)Cl lead to an improvement in the morphology of the surface, providing a more complete and organized structure with slightly higher hydrophobicity.

The antimicrobial tests show antimicrobial activity against drug-resistant and Gram-negative *E. coli* and Gram-positive *S. aureus* and *S. typhimurium*. These results are a second very important advantage of these new materials based on biocompatible PHB.

The third advantage is the growth of physical and mechanical characteristics of materials with the 3 and 5% wt. of Hmi and 1% of Fe(TPP)Cl. The effects of Hmi are certainly higher than those of Fe(TPP)Cl, but for biomedical purposes where an increase in the strength of a non-woven material is not required, Fe(TPP)Cl could be recommended as well.

## 5. Conclusions

The effect of natural and synthetic molecular complexes on the structure and properties of the electrospun composite materials based on PHB was investigated. The possibility of obtaining fibrous materials with high mechanical properties, high antibacterial activity, and controlled wettability was shown in the work. The introduction of 1–5% wt. of hemin and tetraphenylporphyrin with iron has an effect on the supramolecular structure, morphology, and properties of PHB-based fibers due to crystallization processes occurring at the stage of forming and curing of the fiber. The addition of metal atom (trivalent iron) contained in the tetrapyrrole ring of chosen complexes makes it possible to obtain an optimal balance of electrical conductivity and viscosity for forming defect-free uniform fibers. However, the influence of porphyrin complexes on the supramolecular structure had the opposite effect, with similar trends. This observation serves as a basis for the modification and directional design of the supramolecular structure of semi-crystalline polymers and properties of the fibrous material.

## Figures and Tables

**Table 1:** Average values of the characteristics of the fibrous layer of PHB-Hmi and PHB-Fe(TPP)Cl composites.

Sample	Concentration of Additive, %	Density, g/cm^3^ (±S.D., n = 10)	Average Diameter, µm (±S.D., n = 100)	Porosity, % (±S.D., n = 50)
PHB	0	0.30 ± 0.01	3.50 ± 0.08	80 ± 2.0
PHB-Hmi	1	0.20 ± 0.02	2.06 ± 0.07	92 ± 1.5
PHB-Hmi	3	0.20 ± 0.01	1.77 ± 0.04	92 ± 1.5
PHB-Hmi	5	0.17 ± 0.01	1.77 ± 0.04	94 ± 1.2
PHB-Fe(TPP)Cl	1	0.21 ± 0.02	2.07 ± 0.07	93 ± 1.4
PHB-Fe(TPP)Cl	3	0.20 ± 0.02	3.55 ± 0.04	95 ± 1.2
PHB-Fe(TPP)Cl	4	0.16 ± 0.01	3.54 ± 0.04	89 ± 1.2

**Table 2:** Results of the DCS analysis, where χ—crystallinity degree Δ ± 2.5%, ∆H – melting enthalpy Δ ± 2.5%, T_m_—melting temperature Δ ± 2%.

Sample	Concentration of Additive, %	First Heating Run		χ PHB, %	Second Heating Run		χ PHB, %
		T_m_, °C	ΔH, J/g		T_m_, °C	ΔH, J/g	
PHB	0	175	93.1	65.2	170	90.8	63.9
PHB-Hmi	1	172	81.8	57.0	168	78.7	54.9
PHB-Hmi	3	173	77.8	53.1	170	75.4	51.5
PHB-Hmi	5	174	75.3	50.4	170	72.7	48.6
PHB-Fe(TPP)Cl	1	170	92.9	65.5	148	67.4	41.5
PHB-Fe(TPP)Cl	3	169	96.8	68.3	157	73.2	51.6
PHB-Fe(TPP)Cl	5	169	119.0	84.5	156	76.3	53.7

**Table 3:** Results of the mechanical analysis.

Sample	Concentration of Additive, %	Tensile Strength, MPa ±0.02 MPa	Elongation at Break, % ±0.2 %
PHB	0	1.7	3.6
PHB-Hmi	1	0.7	4.7
PHB-Hmi	3	1.9	4.7
PHB-Hmi	5	5.5	6.1
PHB-Fe(TPP)Cl	1	2.1	3.5
PHB-Fe(TPP)Cl	3	1.6	3.5
PHB-Fe(TPP)Cl	5	1.4	3.6

**Table 4:** Results of the biological analysis.

Test Culture	Initial Test Culture, CFU/mL	Sample, CFU/mL	Control, CFU/mL
	PHB with 3% wt. Hmi		
*S. aureus* p 209	2.1 × 10^4^	1.8 × 10^3^	8.6 × 10^3^
*E. coli* 1257	2.0 × 10^4^	<1 × 10^2^	9.8 × 10^3^
*S. typhimurium*	2.0 × 10^4^	2.1 × 10^3^	8.1 × 10^3^
	**PHB with 3% wt. Fe(TPP)Cl**		
*S. aureus* p 209	2.0 × 10^4^	1.8 × 10^3^	4.0 × 10^3^
*E. coli* 1257	2.0 × 10^4^	<1 × 10^2^	9.0 × 10^3^
*S. typhimurium*	2.2 × 10^4^	1.0 × 10^3^	6.0 × 10^3^

## Supplementary Materials

The following supporting information can be downloaded at: https://www.mdpi.com/article/10.3390/jfb13010023/s1, Figure S1: DSC thermograms of PHB-Hmi composites: first heating run (a), second heating run (b) and PHB-Fe(TPP)Cl composites: first heating run (c), second heating run (d.; Figure S2: X-ray diffractograms of PHB-Hmi composites: 0% wt. (a), 1% wt. (b), 3% wt. (c), 5% wt. (d); Figure S3: Small Angle X-ray Scattering of PHB-Hmi composites: 0% wt. (1), 1% wt. (2), 3% wt. (3), 5% wt. (4); Figure S4: EPR spectra of the spin probe TEMPO in structure of samples PHB-Hmi (a) and PHB-Fe(TPP)Cl (b), where 1–0% wt., 2–1% wt., 3–3% wt., 4–5% wt. of the additive; Figure S5: Mechanical tests curves of samples PHB-Hmi (a) and PHB-Fe(TPP)Cl (b), where blue—0% wt., yellow—1% wt., grey—3% wt., red—5% wt. of the additive. Figure S6: Photo (a) and schematic view of the single-capillary laboratory unit for the electrospinning process (b), where: 1— protective installation box; 2—bin with a polymer solution and a capillary; 3—high voltage source; 4—stable precipitating electrode, 5—air pressure regulator.
