# Supplementary material for: The Comparison of Advanced Electrospun Materials Based on Poly(-3-hydroxybutyrate) with Natural and Synthetic Additives
Source: J Funct Biomater. 2022 Feb 28;13(1):23. doi: 10.3390/jfb13010023 (PMC8955504; doi:10.3390/jfb13010023)
Supplement: Supplementary file 1 [file jfb-13-00023-s001.zip › jfb-1604219-supplementary.pdf]

Figure S1. DSC thermograms of PHB-Hmi composites: first heating run (a), second heating run (b) and PHB-Fe(TPP)Cl composites: first heating run (c), second heating run (d).

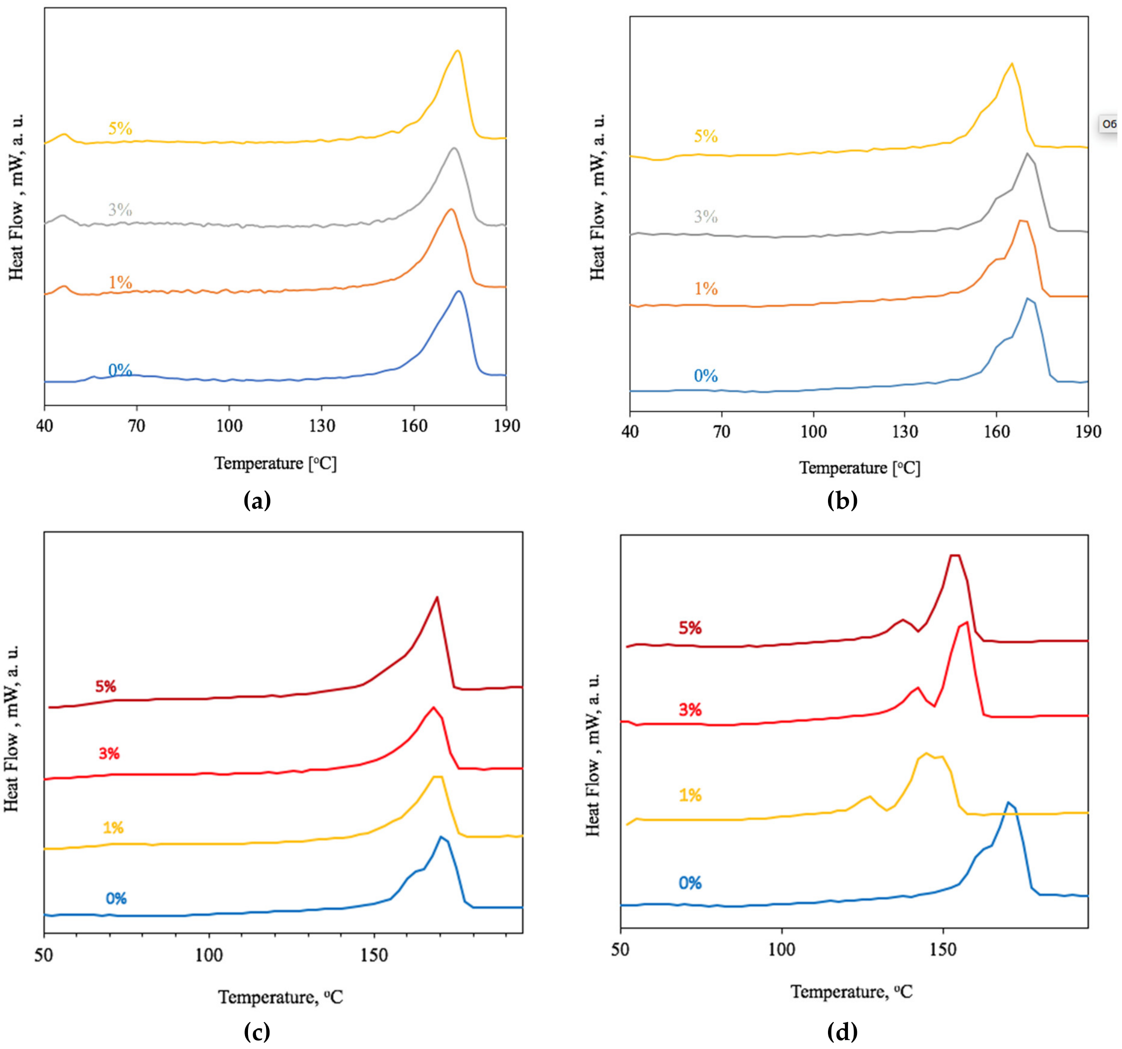

Figure S2. X-ray diffractograms of PHB-Hmi composites: 0% wt. (a), 1 % wt. (**b**), 3 % wt. (**c**), 5 % wt. (**d**).

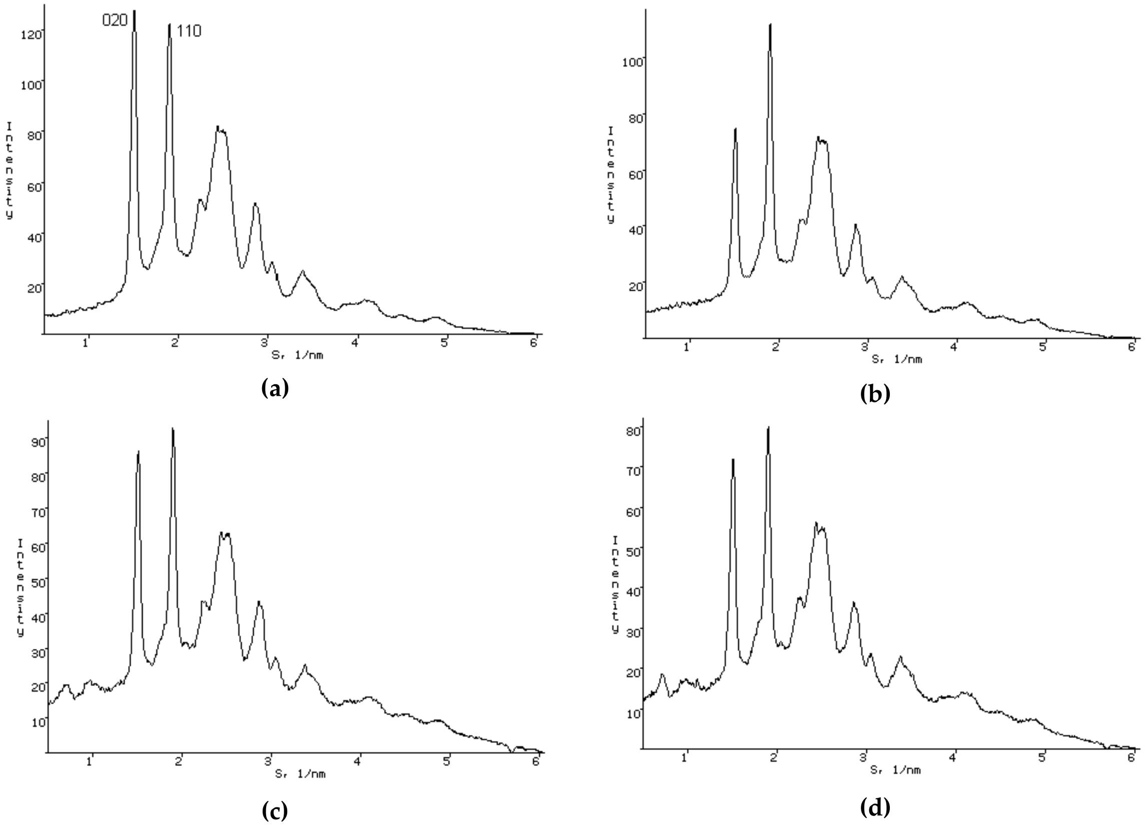

Figure S3. Small Angle X-ray Scattering of PHB-Hmi composites: 0% wt. (1), 1 % wt. (**2**), 3 % wt. (**3**), 5 % wt. (**4**).

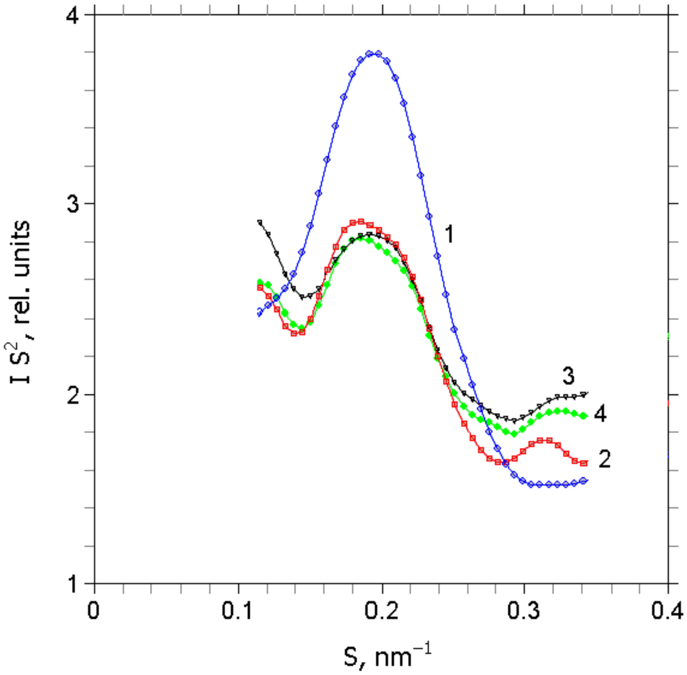

Figure S4. EPR spectra of the spin probe TEMPO in structure of samples PHB-Hmi (**a**) and PHB-Fe(TPP)Cl (**b**), where 1 – 0% wt., 2 – 1% wt., 3 – 3% wt., 4 – 5% wt. of the additive.

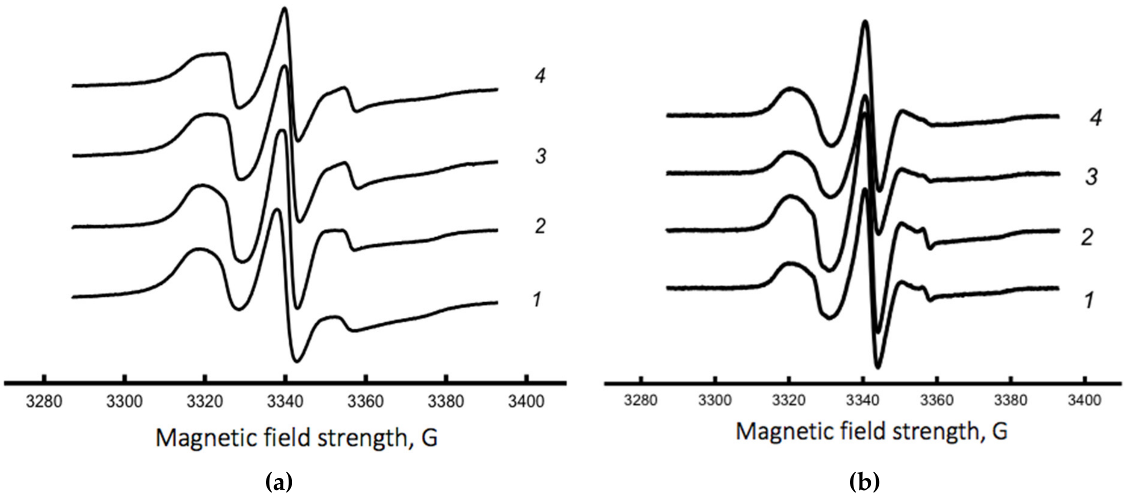

Figure S5. Mechanical tests curves of samples PHB-Hmi (**a**) and PHB-Fe(TPP)Cl (**b**), where blue – 0% wt., yellow – 1% wt., grey – 3% wt., red – 5% wt. of the additive.

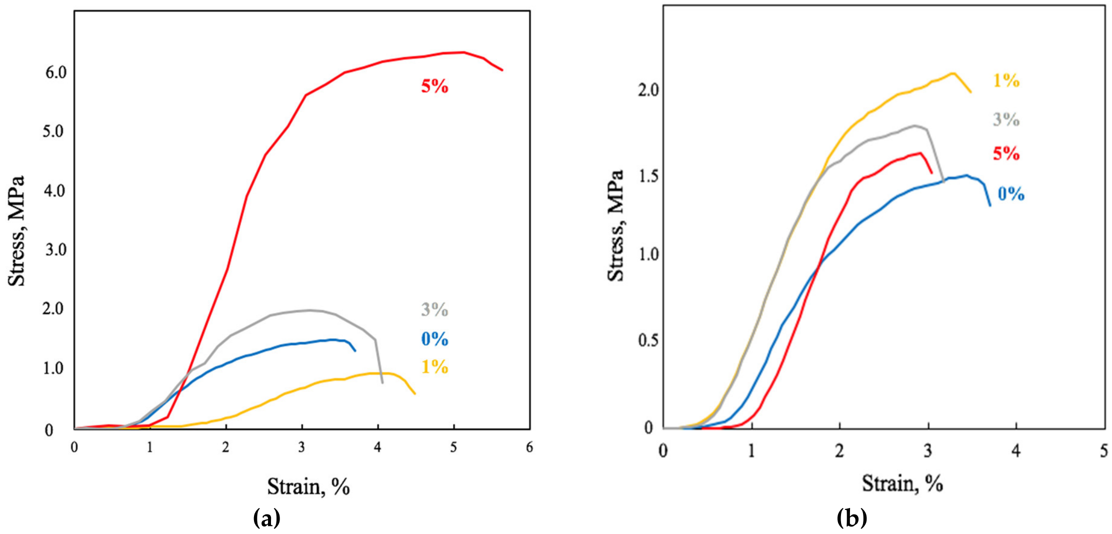

Figure S6. Photo (a) and schematic view of the single-capillary laboratory unit for the electrospinning process (b), where: 1 – protective installation box; 2 – bin with a polymer solution and a capillary; 3 – high voltage source; 4 – stable precipitating electrode, 5 – air pressure regulator.

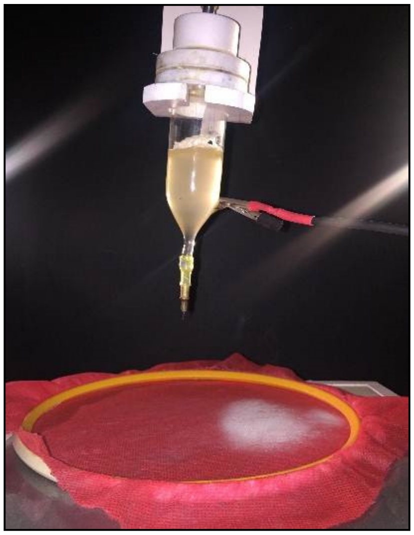

(a)

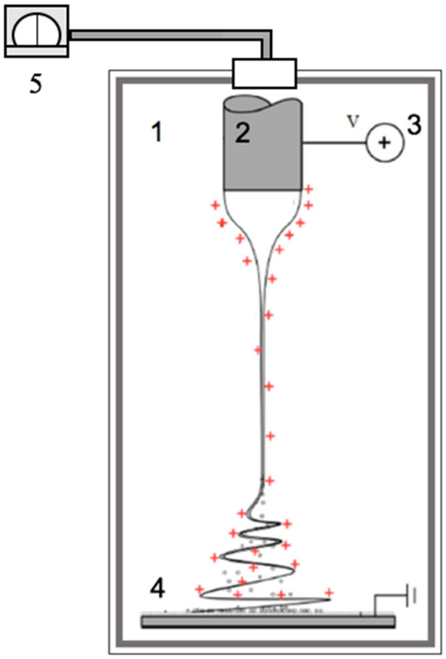

(b)
